# Recent Advances in NMR Protein Structure Prediction with ROSETTA

**DOI:** 10.3390/ijms24097835

**Published:** 2023-04-25

**Authors:** Julia Koehler Leman, Georg Künze

**Affiliations:** 1 Center for Computational Biology, Flatiron Institute, Simons Foundation, New York, NY 10010, USA; julia.koehler.leman@gmail.com; 2 Institute for Drug Discovery, Medical Faculty, University of Leipzig, Brüderstr. 34, D-04103 Leipzig, Germany; 3 Interdisciplinary Center for Bioinformatics, University of Leipzig, Härtelstr. 16-18, D-04107 Leipzig, Germany

**Keywords:** Rosetta, NMR spectroscopy, protein structure prediction, molecular modeling, chemical shifts, residual dipolar couplings, pseudocontact shifts, paramagnetic relaxation enhancements

## Abstract

Nuclear magnetic resonance (NMR) spectroscopy is a powerful method for studying the structure and dynamics of proteins in their native state. For high-resolution NMR structure determination, the collection of a rich restraint dataset is necessary. This can be difficult to achieve for proteins with high molecular weight or a complex architecture. Computational modeling techniques can complement sparse NMR datasets (<1 restraint per residue) with additional structural information to elucidate protein structures in these difficult cases. The Rosetta software for protein structure modeling and design is used by structural biologists for structure determination tasks in which limited experimental data is available. This review gives an overview of the computational protocols available in the Rosetta framework for modeling protein structures from NMR data. We explain the computational algorithms used for the integration of different NMR data types in Rosetta. We also highlight new developments, including modeling tools for data from paramagnetic NMR and hydrogen–deuterium exchange, as well as chemical shifts in CS-Rosetta. Furthermore, strategies are discussed to complement and improve structure predictions made by the current state-of-the-art AlphaFold2 program using NMR-guided Rosetta modeling.

## 1. Introduction

NMR spectroscopy is a powerful method for characterizing protein structures at high resolution in the liquid or solid state. Currently, the Protein Databank (PDB) [1] includes about 7% (>12,400) protein structures and about 11.5% (>1700) nucleic acid structures (DNA and RNA) that were determined by NMR spectroscopy. NMR structure determination typically relies on a large number of structural restraints derived from different NMR data sources, such as atom pair distance restraints, angle restraints, or orientation restraints [2]. Restraints (also called “constraints” in Rosetta jargon) are used for model scoring and imply the use of an energy function. They are used to guide a structure search algorithm, such as simulated annealing, molecular dynamics, or Monte Carlo optimization, producing an ensemble of structures that best satisfy the NMR restraints [3]. However, for large proteins in solution, (>25 kDa) NMR datasets can be sparse because of low signal-to-noise ratios, low spectral resolution, or difficulties in obtaining an unambiguous assignment of the NMR signals [4,5]. For solid-state NMR spectroscopy, protein size is not a problem per se [6], but difficulties can arise, e.g., from peak broadening and difficulties in distinguishing NMR signals owing to intra- vs. intermolecular NMR signals in protein complexes [7,8,9]. In these challenging structure determination cases, computational modeling is necessary to supplement sparse NMR restraint sets with other sources of structural information. While the field of structural biology has been revolutionized by AlphaFold2 [10], predicting de novo structures with high accuracy in many cases [11], NMR data are still needed for structure validation. Furthermore, some types of proteins, e.g., amyloids, disordered proteins, and proteins in low-populated states, cannot be predicted with AlphaFold2 [12,13] but can be studied by NMR spectroscopy [14].

Sophisticated software packages are needed to automate NMR structure generation. Several cycles of spectral assignment, restraint generation, and structure calculation are usually run to resolve ambiguities in the NMR data and to obtain a converged structural ensemble with high precision and accuracy. Some of the most frequently used NMR software packages for biomolecular structure determination include ARIA [15,16], CYANA [17], ASDP [18], Xplor-NIH [19,20], NMRFAM-Sparky [21], and Rosetta [22]. These programs come with additional tools to process and analyze the NMR data prior to the structure calculations and to check the quality of the generated models afterward. To simplify installation, maintenance, and usage of these software tools and to improve the reproducibility of the computational workflows, dedicated software environments such as NMRbox [23] and CCPN [24,25], or web-accessible services such as GeNMR [26], ARIAWeb [27], and PONDEROSA-C/S [28] have been developed.

A component of many NMR toolchains is Rosetta [22,29], which in itself is a compilation of biomolecular modeling algorithms that can calculate physically realistic structural models of proteins and other biomolecules with and without NMR data. Only a few structural biomolecular modeling frameworks have similar capabilities to Rosetta, spanning applications in structure prediction and modeling with experimental data to protein design and small-molecule drug discovery. Rosetta can be used to predict protein structures from sparse NMR data [30,31,32,33,34,35] because the latter is complemented by sophisticated biomolecular modeling algorithms. The use of biomolecular modeling algorithms distinguishes Rosetta from many other NMR programs that rely on the availability of a large number of NMR restraints to obtain a confident structural model. In addition, Rosetta integrates algorithms that use data from several other biophysical experiments, such as electron densities from cryo-electron microscopy (cryo-EM) [36,37,38,39] or X-ray crystallography [40,41], small-angle X-ray scattering (SAXS) [42,43], and mass spectrometry (MS) [44,45,46,47]. This comprehensive toolbox makes it possible to predict structures of large proteins or protein complexes via an integrative structural biology approach. Many of these Rosetta tools can be run via web-accessible servers [48,49,50,51], which facilitates the use of Rosetta by non-specialist users.

In this review, we summarize the NMR tools available in Rosetta, describe the underlying theory and implementation, and explain new tools using NMR data that were introduced in the last six years. We highlight recent studies combining Rosetta with NMR spectroscopy, including integrative structural biology studies on large complexes, and provide a perspective on synergies between NMR-data-guided Rosetta and AlphaFold2, which can be exploited in the future.

## 2. Basic Rosetta Algorithms and Scoring Procedures

While the specific structure calculation approaches used by different Rosetta tools vary, many protocols use a Monte Carlo Metropolis sampling algorithm to efficiently traverse the conformational search space. Backbone and side chain sampling is performed in separate calculation steps, using precomputed peptide fragment or rotamer libraries, respectively, to quickly model the backbone or side chain conformational preferences. Another distinction is made between low-resolution (or “centroid”) and high-resolution (or “full-atom”) modes. In the low-resolution mode, the side chain of each residue is represented by a super atom (“centroid”). This reduces the degrees of freedom that need to be sampled but preserves the chemical features of the amino acid residue. A typical low-resolution sampling protocol involves the folding of the protein-main chain by replacing the existing backbone with a peptide fragment with altered conformation. A fragment denotes a continuous stretch of protein backbone with a structure defined by its ϕ, ψ, and ω torsion angles. In the high-resolution mode, all atoms, including main chain and side chain atoms, are present. A typical sidechain optimization protocol is the Rosetta Packer, which runs a Monte Carlo-simulated annealing protocol to find the combination of sidechain conformations with the lowest energy.

The original Rosetta de novo structure prediction algorithm developed by Simons and Baker [52] predicts 3D structures of proteins by the assembly of short, usually 3- and 9-mer amino acid residue fragments via a Monte Carlo procedure and evaluates models with the Rosetta scoring function. Only the amino acid sequence is needed as input to the de novo folding algorithm. In addition, Rosetta provides other algorithms for different structure prediction tasks. If structures of homologous proteins are available, they can serve as templates for modeling using the Rosetta comparative modeling (RosettaCM) [53] method, usually achieving better accuracy than de novo structure prediction. Moreover, RosettaLigand [54], RosettaDock [55], and Rosetta FlexpPepDock [56] were developed for predicting the structures of protein–ligand, protein–protein, and protein–peptide complexes. All these algorithms can be guided with the help of experimental data. The most recent Rosetta structure prediction methods, trRosetta [57] and RoseTTAFold [58] differ from the classical Rosetta Monte Carlo algorithm and use instead artificial neural networks (ANNs). trRosetta uses a neural network to generate inter-residue distance, angle, and dihedral restraints for an input protein sequence [57]. Quasi-Newton minimization is then used to optimize the conformation of the amino acid chain into a fold consistent with the restraints. The trRosetta calculation is faster and provides more accurate structure predictions than the fragment assembly protocol. Only a few ten to hundred model structures need to be calculated to reach convergence in the prediction, compared to a few thousand trial structures that need to be calculated in the fragment assembly protocol. Structure predictions with even higher accuracies than those possible with trRosetta can be achieved with RoseTTAFold [58]. RoseTTAFold utilizes a network with 1D, 2D, and 3D attention tracks, which communicate sequence, distance, and coordinate information about the protein to each other. RoseTTAFold has been used to predict hundreds of new structures, including those of protein complexes [59].

An integral part of every Rosetta modeling protocol is the Rosetta scoring function. It is a linear combination of score terms that include physics-based and statistically derived potentials from known structures. The score terms describe energy components coming from, e.g., van der Waals interactions, hydrogen bonds, electrostatic interactions, disulfide bonds, residue solvation, and backbone and side chain torsion angle preferences. A detailed review of the current REF2015 Rosetta scoring function was published by Alford et al. [60]. The REF2015 scoring function is compatible with canonical and noncanonical L-α-amino acids, D-α-amino acids, and peptoids. Scoring functions for nucleic acids [61], membrane proteins [62,63,64,65], and carbohydrates [66] have also been added to Rosetta.

## 3. A Brief History of NMR Methods in Rosetta

Rosetta has been used extensively for NMR-data-assisted protein structure prediction. The original RosettaNMR method used backbone chemical shifts (CSs) to find structurally similar peptide fragments in the PDB, which were assembled by a Monte Carlo algorithm guided by nuclear Overhauser effect (NOE) distance restraints [67]. This approach was later extended by Rohl and Baker to use residual dipolar couplings (RDCs) for structure prediction [68]. Meiler and Baker demonstrated that RosettaNMR could be used to predict the structures of small proteins from unassigned NMR spectral data using an iterative cycle of model generation guided by partial NMR peak assignments and spectral reassignment using newly generated models [69]. This approach was later extended with the CS-Rosetta method for structure prediction of larger proteins up to 25 kDa molecular weight from backbone-only CSs, RDCs, and amide NOEs [30,31,32,70]. NMR CS data are highly valuable for fragment selection and for validation of model quality. However, CS-Rosetta is still limited to small proteins owing to computational bottlenecks. Improvements could be achieved by the integration of additional NMR data with orthogonal information content and by using more advanced search algorithms. The size limit was later pushed up to 40 kDa with the help of more powerful computational sampling algorithms [32,33,71,72], such as Resolution-Adapted Structural RECombination (RASREC) [73] or Protein alignments Obtained by Matching Of Nmr Assignments (POMONA) [72], which was used for CS-based comparative modeling of larger proteins.

Additional Rosetta methods were developed for paramagnetic NMR data to take advantage of their value as long-range restraints for protein 3D fold determination. Schmitz et al. [74] combined backbone pseudocontact shift (PCS) data with Rosetta for structure prediction. Yagi et al. [75] extended this approach to PCS datasets from multiple tagging sites, which has been termed GPS-Rosetta, due to the fact that the position of a nucleus can be determined with PCS data collected on three or more tagging sites by triangulation, similar to the global positioning system. Künze et al. [76] generalized and extended this framework to include other paramagnetic NMR data (RDCs induced by alignment by paramagnetic metal ions, paramagnetic relaxation rates) and used them in other structure determination protocols, including protein–protein docking and protein–ligand docking, in addition to de novo folding. Hartlmüller et al. [77] developed a Rosetta method for paramagnetic relaxation enhancements (PREs) caused by paramagnetic cosolute molecules, referred to as solvent PREs (sPREs) (see Section 6.2).

The CS-Rosetta approach has also been used for RNAs (called CS-Rosetta-RNA). Sripakdeevong et al. [78] integrated ^1^H CS data with Rosetta de novo modeling of RNAs. Using a benchmark set of 28 RNA motifs, including 11 blind prediction targets, CS-Rosetta-RNA could recover structures with accuracies of 0.6 to 2.0 Å for 18 RNAs.

In recurring community-wide benchmarks such as the CASD-NMR (Critical Assessment of automated Structure Determination by NMR) experiment [79,80] or the data-assisted modeling category of the CASP (Critical Assessment of protein Structure Prediction) experiment [34,35], Rosetta ranked among the best-performing methods. This demonstrates the strength of Rosetta in combining NMR data with sophisticated biomolecular modeling algorithms.

## 4. Available NMR Data Implementations

**Figure 1:**
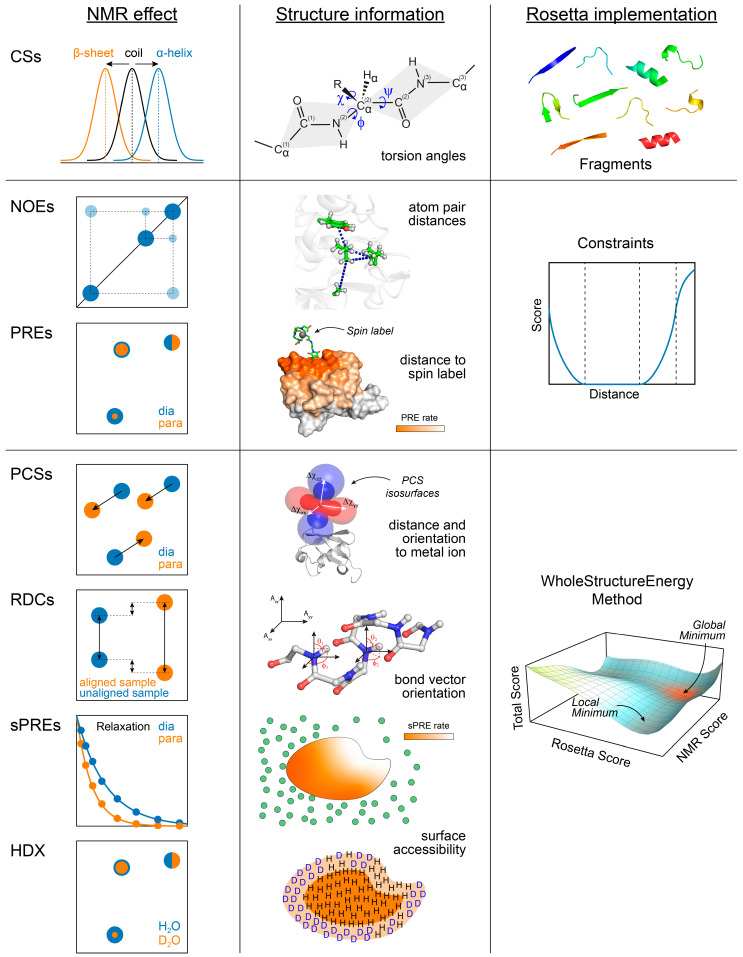
NMR data types that can be used in Rosetta calculations and their algorithmic implementation in Rosetta. The left column schematically depicts the spectral observables used to measure the respective NMR data type. The middle column illustrates the structural information encoded by the NMR data, and the right column shows the sampling or scoring method through which the NMR data are used in Rosetta, as described in the main text.

Figure 1 summarizes the NMR data types, which can currently be used in Rosetta calculations, and depicts their structural information content and the corresponding Rosetta methods. Table 1 gives further details about their algorithmic implementation and lists references where the development and application of these methods were first described.

CSs are sensitive and highly reproducible NMR observables, which provide insights into protein secondary structure and side chain conformations, but also hydrogen bonding, residue solvation, and other parameters [87,88,89]. Isotropic CSs are strongly dependent on the local backbone geometry (i.e., ϕ/ψ angles) and indicative of the secondary structure type [90,91,92], which is the basis for their use in the Rosetta fragment-picking algorithm. The experimental CSs, as well as CS-derived torsion angle and secondary structure predictions, are compared to a database of high-resolution structures to select matching backbone fragments [30,81]. In addition to their use in the fragment selection step, CSs can be used for model validation by augmentation of the Rosetta scoring function. A score term representing the difference between experimental and back-calculated CSs (predicted, e.g., with SPARTA+ [93], SHIFTX2 [94], or PROSHIFT [95]) is used to rescale the Rosetta score to identify models that are biophysically realistic and in agreement with the experimental data. In the case of homology modeling, CSs can be used to supplement sequence information and optimize query-to-template alignment in case of low sequence identities [72], thereby supporting Rosetta comparative modeling (RosettaCM [53]).

For the incorporation of NOEs and CS-derived torsion angles, Rosetta has a flexible restraint system (termed “constraints” in Rosetta jargon). Rosetta allows defining constraints with different geometries (distances, angles, torsions) and different potential functions. To avoid introducing artifacts into structural models caused by erroneous or misassigned NMR data, the weight of different constraints can be adjusted according to their confidence levels. For example, for high-confidence NOE distance constraints, a flat-bottom potential is typically used, whereas, for low-confidence distance constraints, a sigmoidal function is a better choice. The sigmoidal function has a negative score value when the constraint is satisfied but is zero when the distance grows much larger than the defined reference distance. Therefore, large constraint violations (e.g., due to incorrectly assigned NOEs) will not negatively bias the structure calculation. Constraints are defined in a Rosetta-specific, line-based file format (detailed documentation available under: https://rosettacommons.org/docs/latest/rosetta_basics/file_types/constraint-file (accessed on 15 March 2023)). The CS-Rosetta toolbox (https://csrosetta.chemistry.ucsc.edu (accessed on 15 March 2023)) provides scripts that facilitate the conversion of NMR-STAR and other file formats into Rosetta-specific file formats.

For simultaneous, automatic NOESY cross-peak assignment and structure generation, the autoNOE–Rosetta protocol was developed by Lange et al. [96,97]. AutoNOE–Rosetta combines the RASREC protocol for automatic structure calculation with algorithms for automatic NOE assignment, such as network anchoring [98], ambiguous restraints generation [99], restraint combination [98], and structure-dependent and independent peak calibration. Starting from CS assignments and unassigned NOESY peak lists, autoNOE–Rosetta can determine NOE cross-peak assignments and generate structural models without manual user intervention. This integrated approach maximizes the number of structural restraints that can be obtained from the NOE data and ensures the self-consistency of the distance restraints. AutoNOE–Rosetta was found to be quite robust against erroneous NMR data and could generate accurate models even in cases of incomplete NOE peak lists and partially incorrect CS assignments [97].

^1^H-^1^H NOEs provide short-range distance information and are typically combined with long-range restraints obtained from RDCs, PCSs, or PREs that report on the global structure. RDCs encode the orientation of inter-nuclear bond vectors (e.g., N-H, Cα-Hα) with respect to an overall alignment frame. RDCs provide long-range orientational restraints, e.g., on the orientation of secondary structure elements or protein domains in multi-domain proteins [100]. They have been used for structure refinement [101] and de novo structure determination [102,103] in various strategies. Models can be scored with RDCs via a so-called WholeStructureEnergy method in Rosetta. This is a special C++ class in the Rosetta source code used for scoring a model with RDCs, PCSs, sPREs, or other data types. Given a model generated, e.g., in a Monte Carlo trial step, the alignment tensor is calculated by singular value decomposition or least-squares fitting procedures, and the correctness of the structural model is evaluated using the quality of the fit between experimental and back-calculated RDCs. Sparse RDC datasets (with and without CSs) and sparse NOE datasets made structure calculations of proteins up to 25 kDa with Rosetta possible [32].

Paramagnetic relaxation enhancements (PREs) are obtained from the analysis of nuclear spin relaxation rates in samples containing a paramagnetic tag, which is typically site-specifically attached to the protein, and then compared to the diamagnetic reference sample [104]. Similar to NOEs, PREs show an r^−6^ distance dependence, and PRE distance restraints can be used in Rosetta through the constraint system. However, due to the larger magnetic moment of the unpaired electron of the paramagnetic tag, PREs can be detected over longer distances and can complement the short-ranging NOE restraints [104]. Solvent PREs (sPREs) are a special form of PRE data that are obtained in experiments using paramagnetic cosolutes that interact with the protein surface non-covalently [105,106]. sPREs provide qualitative information about residue surface accessibility and the global protein fold and are used in Rosetta via a WholeStructureEnergy method [77] (see Section 6.2).

Pseudocontact shifts (PCSs) are obtained from the chemical shift changes in samples containing a paramagnetic metal ion (e.g., a lanthanide (Tb^3+^, Dy^3+^)) compared to protein samples loaded with a diamagnetic metal (e.g., Lu^3+^). Several metal ion-chelating tags for the site-specific introduction of paramagnetic metal ions into proteins have been developed (see reviews [107,108] and references therein). Furthermore, tagging strategies using noncanonical amino acids and bio-orthogonal labeling reactions (e.g., click chemistry) have been applied [107,109], offering advantages in terms of selectivity and sample stability. However, the preparation of paramagnetically tagged proteins for the measurement of multiple PCS datasets is labor-intensive and requires testing multiple labeling positions to make sure that the protein structure will not be perturbed by the tag.

In contrast to NOEs and PREs, PCSs show an r^−3^ distance dependence and are also dependent on the orientation of the nuclear spin–metal connection vector relative to the frame of the anisotropic magnetic susceptibility (Δχ) tensor of the metal ion [84,110]. By immobilizing the metal ion in the protein, this geometric information can be related to the protein coordinate system, and thus, PCSs can provide a valuable source of structural information. PCSs have facilitated the structure determination in several Rosetta protocols, including de novo folding, protein–protein docking, and protein–ligand docking [74,75,76,85,86].

More recently, hydrogen–deuterium exchange (HDX) data, which are a measure of residue solvent accessibility and local flexibility, were implemented in Rosetta [44,45] (see Section 6.1). Similar to RDCs, PCSs, and sPREs, HDX data are used via a WholeStructureEnergy method.

## 5. Structure Prediction with Chemical Shift Data in Rosetta

CSs are a prerequisite for NMR studies and are obtained in the early stages of a structure determination project. CSs can be converted to the backbone and side chain torsion angle restraints using programs such as TALOS+ [111] and TALOS-N [112], which also provide accurate secondary structure predictions. Due to their high detection sensitivity and structural information content [88,89], CSs are favorable restraints for guiding Rosetta structure prediction. A detailed description of the CS-Rosetta approach can be found in the references [113,114]. Here, we present a short summary of the basic functionalities of CS-Rosetta as well as some recent examples in which CS-Rosetta was crucial for gaining insights into biologically important protein structures.

The original CS-Rosetta method [30] used the fragment picker of the Molecular Fragment Replacement (MFR) method of the NMRPipe software [115]. The MFR method selected fragments from a database of high-resolution structures based on three scores: (1) the chemical shift difference between the target protein and database structure (CS score), (2) the sequence identity between the target and database proteins (Profile score), and (3) the probability of the database ϕ/ψ angles given the target sequence (Rama score). Vernon et al. [81] developed a more advanced and robust CS fragment picker for Rosetta3, which further improved the MFR fragment picker. In addition to the CS, Profile, and Rama scores, two more score terms were added to the current CS fragment picker: (1) the TALOS-SS-similarity score, which evaluates the difference between the CS-derived, TALOS+-predicted [111] secondary structure of a residue in the target protein and the secondary structure of matching residues in the database proteins, and (2) the Phi/Psi-SquareWell score, which is calculated by comparing the CS-derived ϕ/ψ predictions with the ϕ/ψ values of a candidate fragment. These score terms take advantage of the accurate secondary structure and torsion angle predictions from TALOS+ [111] and improve the quality of the CS-Rosetta fragments [81].

CSs are also used in the RosettaCM/POMONA protocol [72], which uses CSs for improving the accuracy of query-to-template alignments within RosettaCM [53] for comparative modeling with multiple template structures. In the low-sequence-identity range, the quality of POMONA alignments was considerably better than the one generated by the sequence-based alignment program HHSearch [116] but not as good as the one from DALI [117], which is designed to find structurally similar proteins, regardless of amino acid sequence. CS-RosettaCM/POMONA calculations were also used for the structure elucidation of membrane proteins [82,83].

CS-Rosetta has become a widely used method in the structural biology community, especially for proteins where only backbone assignments are available, and complete side chain assignments cannot be obtained. Web-accessible servers for CS-Rosetta, hosted, e.g., by the Biological Magnetic Resonance Data Bank (BMRB) (https://csrosetta.bmrb.io/ (accessed on 15 March 2023)) and the Bax group (https://spin.niddk.nih.gov/bax/nmrserver/csrosetta/ (accessed on 15 March 2023)), make the method easy to use by non-specialists. Thus, CS-Rosetta has been a preferred method for structure calculations on membrane proteins or large protein assemblies.

Rosetta NMR structure calculations of membrane proteins can make use of the RosettaMP framework [64], which includes fast-to-calculate scoring functions for membrane environments. The implicit membrane solvent model of RosettaMP captures important properties of biological membranes, e.g., hydrophobic thickness, lipid composition, bilayer anisotropy, and the presence of water-filled pores or holes in membrane proteins [65]. This improves the modeling and design of membrane–protein structural features such as transmembrane helix packing and alignment, the position of aromatic residues at the water–membrane interface, and the presence of polar channels or cavities in membrane proteins that can be permeated by ions or small molecules.

Zhao et al. [118] computed the structure of Aquaporin Z (AqpZ) by CS-Rosetta to investigate its biological mechanism. AqpZ is an integral membrane protein that facilitates the transport of water across *E. coli* cell membranes. The ensemble of AqpZ models was highly converged and revealed that the side chain of the selectivity filter gate residue Arg189 is stabilized in a conformation parallel to the membrane normal by two hydrogen bonds, suggesting that the protein is permanently open under the synthetic membrane composition conditions of the NMR experiment [118].

**Figure 2:**
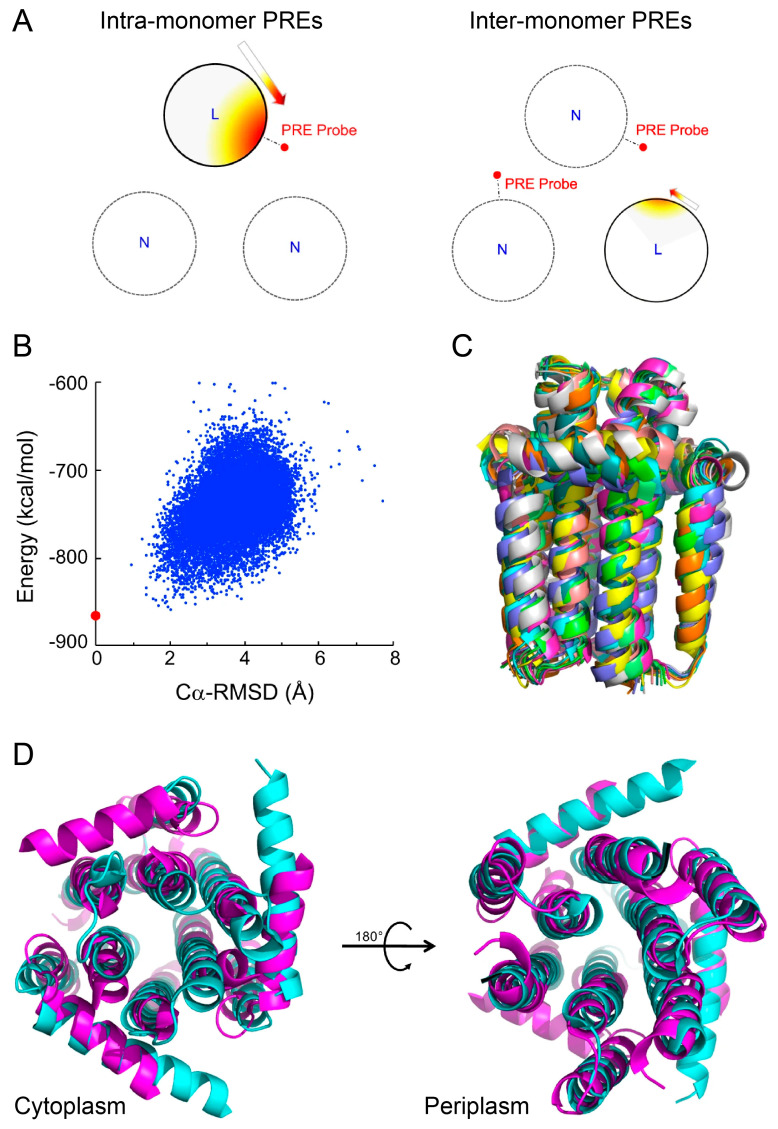
Structure determination of homo-trimeric DgkA in *E. coli* membrane environment using Rosetta. (**A**) Schematic depiction of intra-monomer (left) and inter-monomer PRE measurements (right). Large circles represent transmembrane helices. “L” and “N” are used to mark the isotopically labeled and non-labeled monomers, respectively. (**B**) Folding energy landscape represented by the Rosetta score-vs.-RMSD plot for DgkA. The RMSD is calculated with respect to the lowest energy structure (red dot). (**C**) Ensemble of 10 CS-Rosetta models of DgkA with the best score. (**D**) Comparison of the CS-Rosetta model of DgkA reconstituted in *E. coli* membrane total extracts (magenta) with the one in lipidic cubic phase determined by X-ray crystallography (cyan) (PDB: 3ZE4) [74], viewed from the cytoplasm and periplasm. Reprinted (adapted) with permission from [119]. Creative Commons Attribution 4.0 International License.

Li et al. [119] combined solid-state NMR data and CS-Rosetta calculations to determine the structure of Diacylglycerol kinase (DgkA) in phospholipid bilayers (Figure 2). DgkA is an all-helical, trimeric membrane protein (42 kDa) that is responsible for the ATP-dependent phosphorylation of diacylglycerol to phosphatidic acid. Guided by the CSs as well as PRE data obtained within and between monomers, the Rosetta calculations yielded a well-defined trimeric structure. The structure deviated from the solution structure of DgkA in micelles but was similar to the structure determined by X-ray crystallography (Figure 2) [119]. The study highlights that the membrane mimetic environment has an important influence on the structure of all-helical membrane proteins.

Bender et al. [120] developed models of the peptide hormone ghrelin bound to the growth hormone secretagogue receptor 1a (GHSR), which is a class A G protein-coupled receptor. The authors employed an integrative structure biology approach, combining solid-state NMR spectroscopy, site-directed mutagenesis, and Rosetta modeling. Solid-state NMR CS data obtained on ^13^C-labeled ghrelin in the receptor-bound state were used as backbone restraints in an iterative comparative modeling and flexible peptide docking protocol to develop a model of the ghrelin-GHSR complex. The ensemble of models was validated against mutational data.

## 6. Recent Developments of NMR Modeling Methods in Rosetta

In the following sections, we will describe recent developments in Rosetta that were undertaken to add support for additional NMR data types, such as HDX data as well as NMR data obtained from paramagnetic tags (e.g., PCSs) or paramagnetic cosolutes (e.g., sPREs). In addition, a few landmark studies demonstrating the combination of Rosetta with solid-state NMR data and other types of biophysical data for integrative modeling of larger proteins and protein complexes will be highlighted.

### 6.1. Hydrogen–Deuterium Exchange (HDX)

NMR experiments that measure HDX data offer advantages because they have a higher throughput compared to X-ray crystallography, cryo-EM, or a full panel of NMR experiments required for protein 3D structure determination. HDX data contain information about protein structure [121,122], protein dynamics [123,124], and protein binding sites [125] but have low resolution and are insufficient on their own for full structure determination. HDX NMR experiments provide a map of residue-specific HDX rates which are influenced by regional flexibility and residue solvent exposure at the amide hydrogen position [126]. Computational modeling is needed to generate model structures that can be compared to HDX data. Previous studies [127,128,129,130,131,132] demonstrated that sophisticated sampling methods (such as MD simulations) are needed to match structures to the experimental HDX data, as well as to better understand the factors influencing the HD exchange. HDX data measured from MS have also been used for computational structure prediction [133] and for protein–protein docking [134,135].

**Figure 3:**
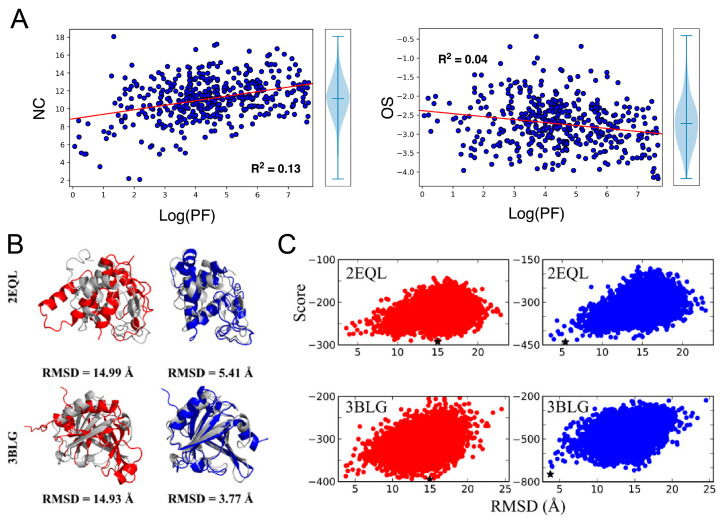
Rosetta structure prediction with HDX data. (**A**) Correlations of the NMR HDX protection factor (PF) with neighbor count (NC) (left) and order score (OS) (right). Linear regression lines are shown in red. Reprinted with permission from [45]. Copyright 2022 Elsevier. (**B**) Lowest-scoring models of protein horse milk lysozyme (PDB: 2EQL) and bovine β-lactoglobulin (PDB: 3BLG) filtered without (red) and with (blue) HDX NMR data are compared to the crystallographic reference structures (gray). (**C**) Score-vs.-RMSD plots of Rosetta models of 2EQL and 3BLG scored without (red) and with (blue) HDX NMR data. Reprinted with permission from [44]. Copyright 2021 American Chemical Society.

Marzolf and coworkers [44] developed a computational methodology to incorporate HDX NMR data into de novo protein structure prediction with Rosetta. The authors introduced a new HDX NMR score term to the Rosetta energy function. The scoring algorithm considers model features that provide estimates of local residue flexibility and solvent exposure. The energy of short-range and long-range backbone–backbone hydrogen bonds (hbond_sr_bb, hbond_lr_bb) and backbone–sidechain hydrogen bonds (hbond_bb_sc), as well as the order score [136,137], were used to quantify residue flexibility. The latter is a Rosetta-calculated score for residue disorder, with higher values indicating higher disorder [137]. They found that lower HDX rates correlate with stronger hydrogen bond energy and lower order score. The other factor affecting the HDX rate is solvent exposure, which was quantified using the amide group neighbor count and relative residue solvent accessible surface area (rSASA). They also found expected correlations, such as decreased amide group HDX rates with increased neighbor atom counts and decreased rSASA. The authors defined the HDX score as a weighted sum of the Rosetta score and the score components for solvent accessibility (neighbor count, rSASA) and regional flexibility (hydrogen bond energy, order score). The individual score components were calculated based on the deviation of the calculated metrics for a Rosetta model relative to the distribution observed in protein X-ray structures. If the exposure or flexibility parameters of a residue’s amide group in a Rosetta model agreed with the distribution of the parameters in the crystal structures, the residue was rewarded using a term-specific scoring function, with those opposite penalized. The performance of the HDX NMR protocol was examined on 38 proteins with available experimental HDX NMR data. The model RMSD to the corresponding crystal structure over the whole benchmark set improved by 1.4 Å on average, including seven proteins with an improvement of greater than 4 Å and one protein with an improvement of more than 11 Å (Figure 3). The model RMSD for core residues improved by 0.9 Å on average, with an improvement as high as 10.5 Å, indicating that the improvement was not only occurring in disordered regions. This study emphasizes that HDX NMR data are highly useful for improving the scoring and selection of models from computational structure prediction in Rosetta.

The previous scoring method by Marzolf [44] used HDX strength categories (i.e., strong, medium, or weak protection) for correlation with structural features instead of actual HDX rates. Nguyen et al. [45] extended the HDX NMR scoring method in Rosetta by using explicit quantitative protection factors (PFs), which report on the HDX rates in the structure calculation. PFs are defined as the ratio of the sequence-dependent intrinsic HDX rate constant to the observed exchange rate constant. Backbone amides with higher PF are expected to be less flexible (i.e., participate strongly in hydrogen bonding) and/or have less solvent exposure. From observed correlations between PFs, residue flexibility, and exposure metrics (Figure 3A), the authors developed a scoring method to predict HDX PFs from structures using linear regression, with the difference between experimental and predicted values incorporated as a score term. Method performance was evaluated on a benchmark set of 10 proteins, and an average RMSD improvement of the selected models of 5.1 Å was observed. The number of cases in which the selected model had an RMSD below 5.5 Å increased from 7/10 without HDX restraints to 9/10 in the presence of HDX restraints.

### 6.2. Paramagnetic NMR

Paramagnetic NMR data (PCSs, PREs, and RDCs in paramagnetically aligned samples) provide valuable structural restraints and are used for many applications in structural biology and drug discovery, which have been reviewed before [84,110,138,139,140,141,142]. Among the three paramagnetic NMR data types, PCSs are particularly useful. They can be detected over long distances (up to 40 Å [143], and with more rigid tags even up to 70 Å [138], due to their r^−3^ dependence), encode additional orientation information, and can be measured with high accuracy from the chemical shift difference between the diamagnetic and paramagnetic NMR spectra. The groups of Otting and Huber first demonstrated the potential of combining PCS restraints with Rosetta [74,75,144]. Künze et al. refactored and generalized Rosetta to include all three paramagnetic NMR data types in one common framework and extended the application range of the framework [76]. It is now possible to use PCSs, RDCs, and PREs together with local NMR restraints (CSs, NOEs) for de novo modeling, comparative modeling, protein–protein and protein–ligand docking, modeling of symmetric complexes, and more tasks.

Solvent PREs (sPREs) were introduced into Rosetta by Hartlmüller et al. [77]. sPREs carry surface accessibility information and can be induced by paramagnetic cosolutes, e.g., Gd^3+^ chelates such as Gd (DTPA-BMA) or nitroxides such as PROXYL derivatives, which are added to the biomolecule sample [106]. The authors presented a protein structure prediction approach in which the distance-to-surface information encoded by the sPRE data is used to assess the correctness of the predicted protein 3D fold [77]. For computational efficiency, a fast-to-compute, grid-based scoring method, in which a trial model is compared to a 3-dimensional grid of sPRE probe positions, was developed. Grid positions that have no spatial overlap with the protein structure are considered accessible to the sPRE probe. The sPRE rate of a protein atom is then predicted by summing the contributions to the PRE rate at the accessible grid points over the integration radius. The sPRE data back-calculated from the model is then compared to the experimental sPRE data using the Spearman correlation coefficient and converted into a score. The sPRE score was found to be a good indicator of model accuracy, especially in the centroid stage of protein structure prediction. Over a wide Cα-RMSD range of 3–20 Å, the sPRE score showed a high correlation with the RMSD value, indicating that it can efficiently evaluate the accuracy of the global fold of a protein, while in the high-resolution range (Cα-RMSD < 2 Å) the Rosetta score showed a better performance. The sPRE data improved conformational sampling and scoring in CS-Rosetta, leading to higher accuracy and convergence in structural models, effectively increasing the size limit of CS-Rosetta. The sPRE-CS-Rosetta method was robust to noisy and sparse sPRE data, which suggests that it can be useful for the structure determination of larger proteins with incomplete resonance assignments and sparse datasets.

The developers of the sPRE-CS-Rosetta method also demonstrated that the application range of sPRE data could be further extended to other biomolecular structure determination tasks. Hartlmüller et al. applied sPRE data to the high-resolution refinement of RNA structures [145] and the detection of transient structures in intrinsically disordered proteins [105], albeit using alternative NMR software packages.

### 6.3. Integrative Structural Biology on Protein Complexes

Pioneering work combining solid-state NMR (ssNMR) spectroscopy, cryo-EM, and Rosetta modeling was conducted by Loquet et al. [146]. Using an integrative structural biology approach, the authors determined the structure of the large *Salmonella typhimurium* type III secretion needle system. Rosetta provided a general framework to integrate the different sources of structural information. A total of 521 NMR distance restraints (359 intrasubunit and 162 intersubunit restraints) were collected from the ^13^C-^13^C ssNMR spectra. Scanning transmission electron microscopy (STEM) measurements and the intersubunit distance restraints indicated that the number of subunits was ~5.7 per helix turn or 11 subunits per two helix turns. Rosetta Fold-and-Dock calculations [147], guided by the helical symmetry of the filament and the intra- and intersubunit ssNMR restraints, yielded a well-converged NMR ensemble with an average pairwise RMSD of 2.1 Å. Two rounds of structure calculations were performed, the first round using only unambiguous axial and intramolecular restraints to resolve some initial ambiguities in the NMR data and the second round using all restraints. Demers et al. [148] applied a similar integrative structural biology approach to the type III secretion needle system from *Shigella flexneri*, using nearly 1000 ssNMR restraints and a 7.7 Å low-resolution cryo-EM density map. Furthermore, low-resolution shape information from SAXS data and sparse NOE and RDC sets were combined with Rosetta modeling by Rossi et al. [42] to elucidate the oligomerization mode of symmetric proteins.

The combination of ssNMR spectroscopy and Rosetta is a favorable combination of methods to obtain the structure of noncrystalline, high-molecular-weight assemblies. Morag et al. [149] demonstrated this for the M13 bacteriophage capsid and could obtain structural models of the repeating unit of the 14 MDa capsid using Rosetta modeling and structural restraints from magic angle spinning (MAS) ssNMR data. In the M13 bacteriophage, the capsid is composed of several thousand identical copies of a major coat protein arranged in a helical array surrounding the core of circular ssDNA. Two-dimensional ^13^C-^13^C CORD and ^13^C-^13^C DARR MAS NMR spectra were used to acquire structural restraints. CS-Rosetta Fold-and-Dock calculations [147] were then used to derive an atomic quaternary structure model of the M13 phage capsid. In total, 95 unambiguous intersubunit restraints and 160 intrasubunit restraints were collected and used in Rosetta modeling. The Rosetta capsid models revealed details of the subunit packing and showed that the capsid consists of stacked pentameric rings with a rise of ~16 Å and a tilt of ~36° between consecutive pentamers. Interestingly, the structure shows that 80 of the 95 intersubunit restraints define a major hydrophobic pocket that is important for stabilizing the subunit packing and that is highly conserved.

More recently, the structures of the large BBSome complex (>400 kDa) [150] and the BAF complex bound to the nucleosome core particle (>1 MDa) [151] were determined using integrative structural modeling with Rosetta. The use of complementary experimental data and Rosetta modeling was key because the resolution of the cryo-EM maps was insufficient to deduce the subunit 3D organization. The subunit structures were obtained by Rosetta comparative or de novo modeling and assembled in the cryo-EM map guided by Rosetta’s electron density score [40,41] and residue pair distance restraints. While the distance restraints used in these studies were derived by cross-linking MS, they are used through the same Rosetta constraint framework as NOE or PRE data. These examples highlight that Rosetta provides high flexibility in combining different types of experimental data and allows for building integrative modeling protocols. The Rosetta-determined BBSome [150] and BAF complex structures [151] could inform on the subunit binding interactions and possible mechanisms of action of disease-related missense mutations.

## 7. Future Directions

The structural biology community has experienced major breakthroughs in highly accurate protein structure prediction in the last two years due to the development of AlphaFold2 [10] and related deep learning methods such as RoseTTAFold [58], ESMFold [152] and OmegaFold [153]. The accuracy that can be reached by those methods is comparable to that of experimental structures in some cases [11]. Surprisingly, for some proteins, the AlphaFold2-generated model was found to match the experimental NMR data as well as or better than the corresponding high-resolution crystal structure [154] or an expert-generated, conventional NMR structure [155]. These results show that AlphaFold2 models can be an accurate representation of the solution conformation of proteins and helpful for guiding the analysis of experimental NMR data. As a result, high-quality protein models are now available that cover the full human proteome [11] and more [156]. Still, there are several synergies between deep learning structure prediction methods and NMR-guided modeling, which can be further exploited.

### 7.1. Augmentation of Deep Learning Methods with NMR Data

One possible direction of development can be the incorporation of NMR data directly into the neural network prediction process. Next to using the NMR data in a sequential manner to validate or post-process computationally predicted models, certain NMR data could also be directly incorporated in the network architectures of RoseTTAFold or AlphaFold2. Recently, Stahl et al. developed AlphaLink [157], a modified version of AlphaFold2 that incorporates MS cross-linking (XL) data into the AlphaFold2 network architecture. The XL contact restraints complement and refine the evolutionary-based contact information, and, in return, the co-evolutionary contacts suppress noisy XL data. AlphaLink offers improved performance compared to AlphaFold2 in cases of challenging targets such as proteins with shallow multiple sequence alignments (MSAs) or multiple conformational states [157]. The authors note that their approach is also applicable to other types of experimental distance information (e.g., NOEs). Moreover, Watson et al. [158] have shown that the RoseTTAFold neural network can be modified for other prediction tasks (e.g., protein design), which could offer the possibility to fine-tune the network using structural information from NMR.

### 7.2. Modeling of Alternative Conformational States

In some cases, AlphaFold2 can deliver structure predictions representing more than one conformational state for the same target protein. Using different strategies for preparing the input information for AlphaFold2, such as subsampling of sequences from the MSA created for the target protein [159], iterative masking of columns in the MSA (by in silico mutation to alanine) [160], or providing template structures in different states [161], allowed generating ensembles of dissimilar models with AlphaFold2. The combination of AlphaFold2 and NMR spectroscopy promises to be a powerful approach for assessing the accuracy and functional relevance of these AlphaFold2 ensembles and for better understanding protein structural dynamics [162]. For instance, NMR relaxation dispersion experiments can report on protein conformational changes occurring on the µs-ms timescale [163]. These methods can deliver CS information on alternative minor states and on the interconversion rate between the ground state and the minor state. The CS data can be used to identify models from the AlphaFold2 ensemble that best represent the weakly populated conformational state. Subsequently, CS-Rosetta calculations can be employed to refine the model to high accuracy, as shown by Fenwick et al. [164]. In addition to CS data, PCSs, and other paramagnetic NMR data [104] could also be used to detect lowly populated states using relaxation dispersion [165] or chemical exchange saturation transfer (CEST) experiments [166]. Pilla et al. demonstrated a Rosetta workflow for modeling conformational changes using sparse PCS datasets obtained on the closed and open forms of the 27 kDa dengue virus serotype 2 NS2B-NS3 protease [144]. Similar workflows can be applied to refine AlphaFold2 models towards a state that reflects the experimental NMR measurements. These protein models can be extremely helpful to obtain insight into the molecular function of the lowly populated conformational states, which often play roles in, e.g., enzyme catalysis, ligand binding, or molecular recognition [167,168].

### 7.3. Modeling of Disordered Proteins and Protein Fibrils

Another area where Rosetta and NMR can meaningfully complement AlphaFold2 is the modeling of structurally disordered protein regions. Within the human proteome, about 30% of regions are disordered [169,170], and they frequently interact with other proteins and function as hubs in protein interaction networks. Protein regions with a low confidence score in AlphaFold2, indicated by low predicted Local Distance Difference Test (pLDDT) scores, are almost always disordered. This makes AlphaFold2’s pLDDT score a rigorous metric for identifying disordered regions in proteins [11,171]. However, the extended chain depiction of low-confidence regions visible in AlphaFold2 models is not an accurate representation of the structure of disordered domains. It is well known from NMR and SAXS measurements that disordered regions can contain many transient conformations and adopt compact states, especially when they undergo liquid/liquid phase separation [172,173]. While in silico modeling of disordered proteins has largely been carried out using MD simulations, some Monte Carlo methods in Rosetta have also been applied to disordered regions. Wang et al. modeled disordered regions by increasing the repulsive interactions and turning off attractive forces between residues in disordered regions and with the rest of the protein [174]. Ferrie et al. used the FloppyTail algorithm [175] to model disordered parts of proteins [176]. The fragment picker was also used to predict the local conformational preference of intrinsically disordered proteins with and without CS information [177].

Some disordered proteins can form amyloid fibrils under certain conditions, representing another challenging prediction case for AlphaFold2 [13]. In particular, AlphaFold2 fails to predict the structural polymorphism that is characteristic of some amyloid-forming proteins, such as the tau protein. The tau fibril structures found in different tau pathologies reveal a diversity of folds, which cannot be reasoned from the protein sequence alone [178,179]. However, structures of amyloid fibrils are accessible for characterization by solid-state NMR spectroscopy, providing restraints for Rosetta structural calculations, as shown for Aβ [180,181,182] and α-synuclein [183].

In summary, NMR-guided Rosetta modeling and AlphaFold2 exhibit synergies, which can be exploited to create powerful method workflows. Structural insights on proteins obtained from these simulations will advance our understanding of their biological functions and provide a basis for modifying protein functions for biotechnological and pharmaceutical applications.

## Figures and Tables

**Table 1:** NMR data types available for protein structure prediction with Rosetta.

NMR Data Type	Implementation Type in Rosetta	References— Original Method	References— Other Examples
CSs	Selection of protein backbone fragments for Rosetta fragment assembly algorithm. Scoring of protein structures by comparison of experimental and back-calculated CSs.	[30,81]	[31,32,33,70]
CSs	Identification of template structures for Rosetta comparative modeling by matching of experimental and back-calculated CS assignments.	[72]	[82,83]
HDX	Scoring of protein structures by comparison of experimental and model-predicted protection factors or HDX strength categories. HDX score is a linear combination of residue flexibility and solvent exposure metrics.	[44,45]	
NOEs/PREs	Rosetta distance constraints with user-defined distance range and potential function. Grouping of constraints into ambiguous distance constraints is possible.	[32,33,67]	[34,35]
PCSs	Scoring of protein structures by comparison of experimental and back-calculated PCSs. Determination of lanthanide position and Δχ tensor via grid search and singular value decomposition or least squares fitting procedure.	[74,75,76]	[84,85,86]
PCSs	Iterative regeneration of a backbone fragment library from models with good fit to PCS data for successive rounds of de novo folding.	[85]	
RDCs	Scoring of protein structures by comparison of experimental and back-calculated RDCs. Determination of alignment tensor by singular value decomposition or least-squares fitting procedure.	[32,33,68]	[42,70,71]
sPREs	Scoring of protein structures by calculating the correlation between experimental and back-calculated sPREs. The predicted sPREs are obtained by r^−6^ summation over all grid positions around a protein structure, which are accessible to the paramagnetic probe.	[77]	

## Data Availability

Data sharing not applicable.

## References

[1] Berman H.M., Westbrook J., Feng Z., Gilliland G., Bhat T.N., Weissig H., Shindyalov I.N., Bourne P.E. (2000). The Protein Data Bank. Nucleic Acids Res..

[2] Driscoll P.C., Roberts G.C.K. (2013). Structure Determination by NMR: Overview. Encyclopedia of Biophysics.

[3] Guntert P. (2011). Calculation of Structures from NMR Restraints. Protein NMR Spectroscopy: Practical Techniques and Applications.

[4] Jiang Y., Kalodimos C.G. (2017). NMR Studies of Large Proteins. J. Mol. Biol..

[5] Danmaliki G.I., Hwang P.M. (2020). Solution NMR Spectroscopy of Membrane Proteins. Biochim. Biophys. Acta (BBA)—Biomembr..

[6] Reif B., Ashbrook S.E., Emsley L., Hong M. (2021). Solid-State NMR Spectroscopy. Nat. Rev. Methods Prim..

[7] Mandala V.S., Williams J.K., Hong M. (2018). Structure and Dynamics of Membrane Proteins from Solid-State NMR. Annu. Rev. Biophys..

[8] Liu J., Wu X., Zeng Y., Hu Z., Lu J. (2023). Solid-State NMR Studies of Amyloids. Structure.

[9] Habenstein B., Loquet A. (2016). Solid-State NMR: An Emerging Technique in Structural Biology of Self-Assemblies. Biophys. Chem..

[10] Jumper J., Evans R., Pritzel A., Green T., Figurnov M., Ronneberger O., Tunyasuvunakool K., Bates R., Žídek A., Potapenko A. (2021). Highly Accurate Protein Structure Prediction with AlphaFold. Nature.

[11] Tunyasuvunakool K., Adler J., Wu Z., Green T., Zielinski M., Žídek A., Bridgland A., Cowie A., Meyer C., Laydon A. (2021). Highly Accurate Protein Structure Prediction for the Human Proteome. Nature.

[12] Ruff K.M., Pappu R.V. (2021). AlphaFold and Implications for Intrinsically Disordered Proteins. J. Mol. Biol..

[13] Pinheiro F., Santos J., Ventura S. (2021). AlphaFold and the Amyloid Landscape. J. Mol. Biol..

[14] Gibbs E.B., Cook E.C., Showalter S.A. (2017). Application of NMR to Studies of Intrinsically Disordered Proteins. Arch. Biochem. Biophys..

[15] Linge J.P., Habeck M., Rieping W., Nilges M. (2003). ARIA: Automated NOE Assignment and NMR Structure Calculation. Bioinformatics.

[16] Rieping W., Habeck M., Bardiaux B., Bernard A., Malliavin T.E., Nilges M. (2007). ARIA2: Automated NOE Assignment and Data Integration in NMR Structure Calculation. Bioinformatics.

[17] Güntert P., Buchner L. (2015). Combined Automated NOE Assignment and Structure Calculation with CYANA. J. Biomol. NMR.

[18] Huang Y.J., Tejero R., Powers R., Montelione G.T. (2006). A Topology-Constrained Distance Network Algorithm for Protein Structure Determination from NOESY Data. Proteins.

[19] Schwieters C.D., Kuszewski J.J., Tjandra N., Clore G.M. (2003). The Xplor-NIH NMR Molecular Structure Determination Package. J. Magn. Reson..

[20] Bermejo G.A., Schwieters C.D. (2018). Protein Structure Elucidation from NMR Data with the Program Xplor-NIH. Methods Mol. Biol..

[21] Lee W., Tonelli M., Markley J.L. (2015). NMRFAM-SPARKY: Enhanced Software for Biomolecular NMR Spectroscopy. Bioinformatics.

[22] Leman J.K., Weitzner B.D., Lewis S.M., Adolf-Bryfogle J., Alam N., Alford R.F., Aprahamian M., Baker D., Barlow K.A., Barth P. (2020). Macromolecular Modeling and Design in Rosetta: Recent Methods and Frameworks. Nat. Methods.

[23] Maciejewski M.W., Schuyler A.D., Gryk M.R., Moraru I.I., Romero P.R., Ulrich E.L., Eghbalnia H.R., Livny M., Delaglio F., Hoch J.C. (2017). NMRbox: A Resource for Biomolecular NMR Computation. Biophys. J..

[24] Vranken W.F., Boucher W., Stevens T.J., Fogh R.H., Pajon A., Llinas M., Ulrich E.L., Markley J.L., Ionides J., Laue E.D. (2005). The CCPN Data Model for NMR Spectroscopy: Development of a Software Pipeline. Proteins Struct. Funct. Bioinform..

[25] Skinner S.P., Fogh R.H., Boucher W., Ragan T.J., Mureddu L.G., Vuister G.W. (2016). CcpNmr AnalysisAssign: A Flexible Platform for Integrated NMR Analysis. J. Biomol. NMR.

[26] Berjanskii M., Tang P., Liang J., Cruz J.A., Zhou J., Zhou Y., Bassett E., MacDonell C., Lu P., Lin G. (2009). GeNMR: A Web Server for Rapid NMR-Based Protein Structure Determination. Nucleic Acids Res..

[27] Allain F., Mareuil F., Ménager H., Nilges M., Bardiaux B. (2020). ARIAweb: A Server for Automated NMR Structure Calculation. Nucleic Acids Res..

[28] Lee W., Stark J.L., Markley J.L. (2014). PONDEROSA-C/S: Client–Server Based Software Package for Automated Protein 3D Structure Determination. J. Biomol. NMR.

[29] Bender B.J., Cisneros A., Duran A.M., Finn J.A., Fu D., Lokits A.D., Mueller B.K., Sangha A.K., Sauer M.F., Sevy A.M. (2016). Protocols for Molecular Modeling with Rosetta3 and RosettaScripts. Biochemistry.

[30] Shen Y., Lange O., Delaglio F., Rossi P., Aramini J.M., Liu G.H., Eletsky A., Wu Y.B., Singarapu K.K., Lemak A. (2008). Consistent Blind Protein Structure Generation from NMR Chemical Shift Data. Proc. Natl. Acad. Sci. USA.

[31] Shen Y., Vernon R., Baker D., Bax A. (2009). De Novo Protein Structure Generation from Incomplete Chemical Shift Assignments. J. Biomol. NMR.

[32] Raman S., Lange O.F., Rossi P., Tyka M., Wang X., Aramini J., Liu G., Ramelot T.A., Eletsky A., Szyperski T. (2010). NMR Structure Determination for Larger Proteins Using Backbone-Only Data. Science.

[33] Lange O.F., Rossi P., Sgourakis N.G., Song Y., Lee H.W., Aramini J.M., Ertekin A., Xiao R., Acton T.B., Montelione G.T. (2012). Determination of Solution Structures of Proteins up to 40 KDa Using CS-Rosetta with Sparse NMR Data from Deuterated Samples. Proc. Natl. Acad. Sci. USA.

[34] Ovchinnikov S., Park H., Kim D.E., Liu Y., Wang R.Y., Baker D. (2016). Structure Prediction Using Sparse Simulated NOE Restraints with Rosetta in CASP11. Proteins.

[35] Kuenze G., Meiler J. (2019). Protein Structure Prediction Using Sparse NOE and RDC Restraints with Rosetta in CASP13. Proteins.

[36] Wang R.Y., Kudryashev M., Li X., Egelman E.H., Basler M., Cheng Y., Baker D., DiMaio F. (2015). De Novo Protein Structure Determination from Near-Atomic-Resolution Cryo-EM Maps. Nat. Methods.

[37] DiMaio F., Song Y., Li X., Brunner M.J., Xu C., Conticello V., Egelman E., Marlovits T.C., Cheng Y., Baker D. (2015). Atomic-Accuracy Models from 4.5-Å Cryo-Electron Microscopy Data with Density-Guided Iterative Local Refinement. Nat. Methods.

[38] Wang R.Y., Song Y., Barad B.A., Cheng Y., Fraser J.S., DiMaio F. (2016). Automated Structure Refinement of Macromolecular Assemblies from Cryo-EM Maps Using Rosetta. eLife.

[39] Frenz B., Walls A.C., Egelman E.H., Veesler D., DiMaio F. (2017). RosettaES: A Sampling Strategy Enabling Automated Interpretation of Difficult Cryo-EM Maps. Nat. Methods.

[40] DiMaio F., Terwilliger T.C., Read R.J., Wlodawer A., Oberdorfer G., Wagner U., Valkov E., Alon A., Fass D., Axelrod H.L. (2011). Improved Molecular Replacement by Density- and Energy-Guided Protein Structure Optimization. Nature.

[41] DiMaio F., Echols N., Headd J.J., Terwilliger T.C., Adams P.D., Baker D. (2013). Improved Low-Resolution Crystallographic Refinement with Phenix and Rosetta. Nat. Methods.

[42] Rossi P., Shi L., Liu G., Barbieri C.M., Lee H.W., Grant T.D., Luft J.R., Xiao R., Acton T.B., Snell E.H. (2015). A Hybrid NMR/SAXS-Based Approach for Discriminating Oligomeric Protein Interfaces Using Rosetta. Proteins.

[43] Sønderby P., Rinnan Å., Madsen J.J., Harris P., Bukrinski J.T., Peters G.H.J. (2017). Small-Angle X-Ray Scattering Data in Combination with RosettaDock Improves the Docking Energy Landscape. J. Chem. Inf. Model..

[44] Marzolf D.R., Seffernick J.T., Lindert S. (2021). Protein Structure Prediction from NMR Hydrogen–Deuterium Exchange Data. J. Chem. Theory Comput..

[45] Nguyen T.T., Marzolf D.R., Seffernick J.T., Heinze S., Lindert S. (2022). Protein Structure Prediction Using Residue-Resolved Protection Factors from Hydrogen-Deuterium Exchange NMR. Structure.

[46] Aprahamian M.L., Chea E.E., Jones L.M., Lindert S. (2018). Rosetta Protein Structure Prediction from Hydroxyl Radical Protein Footprinting Mass Spectrometry Data. Anal. Chem..

[47] Drake Z.C., Seffernick J.T., Lindert S. (2022). Protein Complex Prediction Using Rosetta, AlphaFold, and Mass Spectrometry Covalent Labeling. Nat. Commun..

[48] Kim D.E., Chivian D., Baker D. (2004). Protein Structure Prediction and Analysis Using the Robetta Server. Nucleic Acids Res..

[49] London N., Raveh B., Cohen E., Fathi G., Schueler-Furman O. (2011). Rosetta FlexPepDock Web Server—High Resolution Modeling of Peptide–Protein Interactions. Nucleic Acids Res..

[50] Moretti R., Lyskov S., Das R., Meiler J., Gray J.J. (2018). Web-Accessible Molecular Modeling with Rosetta: The Rosetta Online Server That Includes Everyone (ROSIE). Protein Sci..

[51] Du Z., Su H., Wang W., Ye L., Wei H., Peng Z., Anishchenko I., Baker D., Yang J. (2021). The TrRosetta Server for Fast and Accurate Protein Structure Prediction. Nat. Protoc..

[52] Simons K.T., Kooperberg C., Huang E., Baker D. (1997). Assembly of Protein Tertiary Structures from Fragments with Similar Local Sequences Using Simulated Annealing and Bayesian Scoring Functions. J. Mol. Biol..

[53] Song Y., DiMaio F., Wang R.Y., Kim D., Miles C., Brunette T., Thompson J., Baker D. (2013). High-Resolution Comparative Modeling with RosettaCM. Structure.

[54] Meiler J., Baker D. (2006). ROSETTALIGAND: Protein-Small Molecule Docking with Full Side-Chain Flexibility. Proteins.

[55] Gray J.J., Moughon S., Wang C., Schueler-Furman O., Kuhlman B., Rohl C.A., Baker D. (2003). Protein-Protein Docking with Simultaneous Optimization of Rigid-Body Displacement and Side-Chain Conformations. J. Mol. Biol..

[56] Raveh B., London N., Schueler-Furman O. (2010). Sub-Angstrom Modeling of Complexes between Flexible Peptides and Globular Proteins. Proteins.

[57] Yang J., Anishchenko I., Park H., Peng Z., Ovchinnikov S., Baker D. (2020). Improved Protein Structure Prediction Using Predicted Interresidue Orientations. Proc. Natl. Acad. Sci. USA.

[58] Baek M., DiMaio F., Anishchenko I., Dauparas J., Ovchinnikov S., Lee G.R., Wang J., Cong Q., Kinch L.N., Schaeffer R.D. (2021). Accurate Prediction of Protein Structures and Interactions Using a Three-Track Neural Network. Science.

[59] Humphreys I.R., Pei J., Baek M., Krishnakumar A., Anishchenko I., Ovchinnikov S., Zhang J., Ness T.J., Banjade S., Bagde S.R. (2021). Computed Structures of Core Eukaryotic Protein Complexes. Science.

[60] Alford R.F., Leaver-Fay A., Jeliazkov J.R., O’Meara M.J., DiMaio F.P., Park H., Shapovalov M.V., Renfrew P.D., Mulligan V.K., Kappel K. (2017). The Rosetta All-Atom Energy Function for Macromolecular Modeling and Design. J. Chem. Theory Comput..

[61] Chou F.-C., Kladwang W., Kappel K., Das R. (2016). Blind Tests of RNA Nearest-Neighbor Energy Prediction. Proc. Natl. Acad. Sci. USA.

[62] Yarov-Yarovoy V., Schonbrun J., Baker D. (2006). Multipass Membrane Protein Structure Prediction Using Rosetta. Proteins.

[63] Barth P., Schonbrun J., Baker D. (2007). Toward High-Resolution Prediction and Design of Transmembrane Helical Protein Structures. Proc. Natl. Acad. Sci. USA.

[64] Alford R.F., Leman J.K., Weitzner B.D., Duran A.M., Tilley D.C., Elazar A., Gray J.J. (2015). An Integrated Framework Advancing Membrane Protein Modeling and Design. PLoS Comput. Biol..

[65] Alford R.F., Fleming P.J., Fleming K.G., Gray J.J. (2020). Protein Structure Prediction and Design in a Biologically Realistic Implicit Membrane. Biophys. J..

[66] Labonte J.W., Adolf-Bryfogle J., Schief W.R., Gray J.J. (2017). Residue-Centric Modeling and Design of Saccharide and Glycoconjugate Structures. J. Comput. Chem..

[67] Bowers P.M., Strauss C.E.M., Baker D. (2000). Denovo Protein Structure Determination Using Sparse NMR Data. J. Biomol. NMR.

[68] Rohl C.A., Baker D. (2002). De Novo Determination of Protein Backbone Structure from Residual Dipolar Couplings Using Rosetta. J. Am. Chem. Soc..

[69] Meiler J., Baker D. (2003). Rapid Protein Fold Determination Using Unassigned NMR Data. Proc. Natl. Acad. Sci. USA.

[70] Sgourakis N.G., Lange O.F., DiMaio F., Andre I., Fitzkee N.C., Rossi P., Montelione G.T., Bax A., Baker D. (2011). Determination of the Structures of Symmetric Protein Oligomers from NMR Chemical Shifts and Residual Dipolar Couplings. J. Am. Chem. Soc..

[71] Thompson J.M., Sgourakis N.G., Liu G., Rossi P., Tang Y., Mills J.L., Szyperski T., Montelione G.T., Baker D. (2012). Accurate Protein Structure Modeling Using Sparse NMR Data and Homologous Structure Information. Proc. Natl. Acad. Sci. USA.

[72] Shen Y., Bax A. (2015). Homology Modeling of Larger Proteins Guided by Chemical Shifts. Nat. Methods.

[73] Lange O.F., Baker D. (2012). Resolution-Adapted Recombination of Structural Features Significantly Improves Sampling in Restraint-Guided Structure Calculation. Proteins Struct. Funct. Bioinform..

[74] Schmitz C., Vernon R., Otting G., Baker D., Huber T. (2012). Protein Structure Determination from Pseudocontact Shifts Using ROSETTA. J. Mol. Biol..

[75] Yagi H., Pilla K.B., Maleckis A., Graham B., Huber T., Otting G. (2013). Three-Dimensional Protein Fold Determination from Backbone Amide Pseudocontact Shifts Generated by Lanthanide Tags at Multiple Sites. Structure.

[76] Kuenze G., Bonneau R., Leman J.K., Meiler J. (2019). Integrative Protein Modeling in RosettaNMR from Sparse Paramagnetic Restraints. Structure.

[77] Hartlmüller C., Göbl C., Madl T. (2016). Prediction of Protein Structure Using Surface Accessibility Data. Angew. Chem. Int. Ed..

[78] Sripakdeevong P., Cevec M., Chang A.T., Erat M.C., Ziegeler M., Zhao Q., Fox G.E., Gao X., Kennedy S.D., Kierzek R. (2014). Structure Determination of Noncanonical RNA Motifs Guided by 1H NMR Chemical Shifts. Nat. Methods.

[79] Rosato A., Bagaria A., Baker D., Bardiaux B., Cavalli A., Doreleijers J.F., Giachetti A., Guerry P., Güntert P., Herrmann T. (2009). CASD-NMR: Critical Assessment of Automated Structure Determination by NMR. Nat. Methods.

[80] Rosato A., Vranken W., Fogh R.H., Ragan T.J., Tejero R., Pederson K., Lee H.-W., Prestegard J.H., Yee A., Wu B. (2015). The Second Round of Critical Assessment of Automated Structure Determination of Proteins by NMR: CASD-NMR-2013. J. Biomol. NMR.

[81] Vernon R., Shen Y., Baker D., Lange O.F. (2013). Improved Chemical Shift Based Fragment Selection for CS-Rosetta Using Rosetta3 Fragment Picker. J. Biomol. NMR.

[82] Zhang X., Zhang Y., Tang S., Ma S., Shen Y., Chen Y., Tong Q., Li Y., Yang J. (2021). Hydrophobic Gate of Mechanosensitive Channel of Large Conductance in Lipid Bilayers Revealed by Solid-State NMR Spectroscopy. J. Phys. Chem. B.

[83] Ye Y., Tyndall E.R., Bui V., Tang Z., Shen Y., Jiang X., Flanagan J.M., Wang H.-G., Tian F. (2021). An N-Terminal Conserved Region in Human Atg3 Couples Membrane Curvature Sensitivity to Conjugase Activity during Autophagy. Nat. Commun..

[84] Koehler J., Meiler J. (2011). Expanding the Utility of NMR Restraints with Paramagnetic Compounds: Background and Practical Aspects. Prog. Nucl. Magn. Reson. Spectrosc..

[85] Pilla K.B., Otting G., Huber T. (2016). Pseudocontact Shift-Driven Iterative Resampling for 3D Structure Determinations of Large Proteins. J. Mol. Biol..

[86] Chen W.N., Nitsche C., Pilla K.B., Graham B., Huber T., Klein C.D., Otting G. (2016). Sensitive NMR Approach for Determining the Binding Mode of Tightly Binding Ligand Molecules to Protein Targets. J. Am. Chem. Soc..

[87] Wagner G., Pardi A., Wuethrich K. (1983). Hydrogen Bond Length and Proton NMR Chemical Shifts in Proteins. J. Am. Chem. Soc..

[88] Mielke S.P., Krishnan V.V. (2009). Characterization of Protein Secondary Structure from NMR Chemical Shifts. Prog. Nucl. Magn. Reson. Spectrosc..

[89] Wishart D.S. (2011). Interpreting Protein Chemical Shift Data. Prog. Nucl. Magn. Reson. Spectrosc..

[90] Wishart D.S., Sykes B.D., Richards F.M. (1992). The Chemical Shift Index: A Fast and Simple Method for the Assignment of Protein Secondary Structure through NMR Spectroscopy. Biochemistry.

[91] Wishart D.S., Sykes B.D. (1994). The 13C Chemical-Shift Index: A Simple Method for the Identification of Protein Secondary Structure Using 13C Chemical-Shift Data. J. Biomol. NMR.

[92] Wishart D.S., Bigam C.G., Holm A., Hodges R.S., Sykes B.D. (1995). 1H, 13C, and 15N Random Coil NMR Chemical Shifts of the Common Amino Acids. I. Investigations of Nearest-Neighbor Effects. J. Biomol. NMR.

[93] Shen Y., Bax A. (2010). SPARTA+: A Modest Improvement in Empirical NMR Chemical Shift Prediction by Means of an Artificial Neural Network. J. Biomol. NMR.

[94] Han B., Liu Y., Ginzinger S.W., Wishart D.S. (2011). SHIFTX2: Significantly Improved Protein Chemical Shift Prediction. J. Biomol. NMR.

[95] Meiler J. (2003). PROSHIFT: Protein Chemical Shift Prediction Using Artificial Neural Networks. J. Biomol. NMR.

[96] Lange O.F. (2014). Automatic NOESY Assignment in CS-RASREC-Rosetta. J. Biomol. NMR.

[97] Zhang Z., Porter J., Tripsianes K., Lange O.F. (2014). Robust and Highly Accurate Automatic NOESY Assignment and Structure Determination with Rosetta. J. Biomol. NMR.

[98] Herrmann T., Güntert P., Wüthrich K. (2002). Protein NMR Structure Determination with Automated NOE Assignment Using the New Software CANDID and the Torsion Angle Dynamics Algorithm DYANA. J. Mol. Biol..

[99] Nilges M., Macias M.J., O’Donoghue S.I., Oschkinat H. (1997). Automated NOESY Interpretation with Ambiguous Distance Restraints: The Refined NMR Solution Structure of the Pleckstrin Homology Domain from b-Spectrin. J. Mol. Biol..

[100] Chen K., Tjandra N., Zhu G. (2012). The Use of Residual Dipolar Coupling in Studying Proteins by NMR. NMR of Proteins and Small Biomolecules.

[101] Tjandra N., Omichinski J.G., Gronenborn A.M., Clore G.M., Bax A. (1997). Use of Dipolar 1H-15N and 1H-13C Couplings in the Structure Determination of Magnetically Oriented Macromolecules in Solution. Nat. Struct. Biol..

[102] Hus J.-C., Marion D., Blackledge M. (2000). De Novo Determination of Protein Structure by NMR Using Orientational and Long-Range Order Restraints. J. Mol. Biol..

[103] Hus J.C., Marion D., Blackledge M. (2001). Determination of Protein Backbone Structure Using Only Residual Dipolar Couplings. J. Am. Chem. Soc..

[104] Clore G.M., Iwahara J. (2009). Theory, Practice, and Applications of Paramagnetic Relaxation Enhancement for the Characterization of Transient Low-Population States of Biological Macromolecules and Their Complexes. Chem. Rev..

[105] Hartlmüller C., Spreitzer E., Göbl C., Falsone F., Madl T. (2019). NMR Characterization of Solvent Accessibility and Transient Structure in Intrinsically Disordered Proteins. J. Biomol. NMR.

[106] Lenard A.J., Mulder F.A.A., Madl T. (2022). Solvent Paramagnetic Relaxation Enhancement as a Versatile Method for Studying Structure and Dynamics of Biomolecular Systems. Prog. Nucl. Magn. Reson. Spectrosc..

[107] Miao Q., Nitsche C., Orton H., Overhand M., Otting G., Ubbink M. (2022). Paramagnetic Chemical Probes for Studying Biological Macromolecules. Chem. Rev..

[108] Joss D., Häussinger D. (2019). Design and Applications of Lanthanide Chelating Tags for Pseudocontact Shift NMR Spectroscopy with Biomacromolecules. Prog. Nucl. Magn. Reson. Spectrosc..

[109] Widder P., Berner F., Summerer D., Drescher M. (2019). Double Nitroxide Labeling by Copper-Catalyzed Azide–Alkyne Cycloadditions with Noncanonical Amino Acids for Electron Paramagnetic Resonance Spectroscopy. ACS Chem. Biol..

[110] Otting G. (2010). Protein NMR Using Paramagnetic Ions. Annu. Rev. Biophys..

[111] Shen Y., Delaglio F., Cornilescu G., Bax A. (2009). TALOS+: A Hybrid Method for Predicting Protein Backbone Torsion Angles from NMR Chemical Shifts. J. Biomol. NMR.

[112] Shen Y., Bax A. (2013). Protein Backbone and Sidechain Torsion Angles Predicted from NMR Chemical Shifts Using Artificial Neural Networks. J. Biomol. NMR.

[113] Nerli S., Sgourakis N.G. (2019). CS-ROSETTA. Methods Enzymol..

[114] Nerli S., McShan A.C., Sgourakis N.G. (2018). Chemical Shift-Based Methods in NMR Structure Determination. Prog. Nucl. Magn. Reson. Spectrosc..

[115] Delaglio F., Grzesiek S., Vuister G.W., Zhu G., Pfeifer J., Bax A. (1995). NMRPipe: A Multidimensional Spectral Processing System Based on UNIX Pipes. J. Biomol. NMR.

[116] Söding J. (2005). Protein Homology Detection by HMM–HMM Comparison. Bioinformatics.

[117] Holm L. (2020). DALI and the Persistence of Protein Shape. Protein Sci..

[118] Zhao Y., Xie H., Wang L., Shen Y., Chen W., Song B., Zhang Z., Zheng A., Lin Q., Fu R. (2018). Gating Mechanism of Aquaporin Z in Synthetic Bilayers and Native Membranes Revealed by Solid-State NMR Spectroscopy. J. Am. Chem. Soc..

[119] Li J., Shen Y., Chen Y., Zhang Z., Ma S., Wan Q., Tong Q., Glaubitz C., Liu M., Yang J. (2021). Structure of Membrane Diacylglycerol Kinase in Lipid Bilayers. Commun. Biol..

[120] Bender B.J., Vortmeier G., Ernicke S., Bosse M., Kaiser A., Els-Heindl S., Krug U., Beck-Sickinger A., Meiler J., Huster D. (2019). Structural Model of Ghrelin Bound to Its G Protein-Coupled Receptor. Structure.

[121] Frieden C., Hoeltzli S.D., Ropson I.J. (1993). NMR and Protein Folding: Equilibrium and Stopped-Flow Studies. Protein Sci..

[122] Vilar M., Wang L., Riek R., Sigurdsson E.M., Calero M., Gasset M. (2012). Structural Studies of Amyloids by Quenched Hydrogen–Deuterium Exchange by NMR. Amyloid Proteins: Methods and Protocols.

[123] Olofsson A., Sauer-Eriksson A.E., Öhman A. (2009). Amyloid Fibril Dynamics Revealed by Combined Hydrogen/Deuterium Exchange and Nuclear Magnetic Resonance. Anal. Biochem..

[124] Ahmed A.H., Ptak C.P., Fenwick M.K., Hsieh C.-L., Weiland G.A., Oswald R.E. (2013). Dynamics of Cleft Closure of the GluA2 Ligand-Binding Domain in the Presence of Full and Partial Agonists Revealed by Hydrogen-Deuterium Exchange. J. Biol. Chem..

[125] Dyson H.J., Kostic M., Liu J., Martinez-Yamout M.A. (2008). Hydrogen–Deuterium Exchange Strategy for Delineation of Contact Sites in Protein Complexes. FEBS Lett..

[126] Yagi-Utsumi M., Chandak M.S., Yanaka S., Hiranyakorn M., Nakamura T., Kato K., Kuwajima K. (2020). Residual Structure of Unfolded Ubiquitin as Revealed by Hydrogen/Deuterium-Exchange 2D NMR. Biophys. J..

[127] Hilser V.J., Freire E. (1996). Structure-Based Calculation of the Equilibrium Folding Pathway of Proteins. Correlation with Hydrogen Exchange Protection Factors. J. Mol. Biol..

[128] Best R.B., Vendruscolo M. (2006). Structural Interpretation of Hydrogen Exchange Protection Factors in Proteins: Characterization of the Native State Fluctuations of CI2. Structure.

[129] McAllister R.G., Konermann L. (2015). Challenges in the Interpretation of Protein H/D Exchange Data: A Molecular Dynamics Simulation Perspective. Biochemistry.

[130] Petruk A.A., Defelipe L.A., Limardo R.G.R., Bucci H., Marti M., Turjanski A.G. (2013). Molecular Dynamics Simulations Provide Atomistic Insight into Hydrogen Exchange Mass Spectrometry Experiments. J. Chem. Theory Comput..

[131] Mohammadiarani H., Shaw V.S., Neubig R.R., Vashisth H. (2018). Interpreting Hydrogen–Deuterium Exchange Events in Proteins Using Atomistic Simulations: Case Studies on Regulators of G-Protein Signaling Proteins. J. Phys. Chem. B.

[132] Martens C., Shekhar M., Lau A.M., Tajkhorshid E., Politis A. (2019). Integrating Hydrogen–Deuterium Exchange Mass Spectrometry with Molecular Dynamics Simulations to Probe Lipid-Modulated Conformational Changes in Membrane Proteins. Nat. Protoc..

[133] Tran M.H., Schoeder C.T., Schey K.L., Meiler J. (2022). Computational Structure Prediction for Antibody-Antigen Complexes from Hydrogen-Deuterium Exchange Mass Spectrometry: Challenges and Outlook. Front. Immunol..

[134] Pandit D., Tuske S.J., Coales S.J., E S.Y., Liu A., Lee J.E., Morrow J.A., Nemeth J.F., Hamuro Y. (2012). Mapping of Discontinuous Conformational Epitopes by Amide Hydrogen/Deuterium Exchange Mass Spectrometry and Computational Docking. J. Mol. Recognit..

[135] Roberts V.A., Pique M.E., Hsu S., Li S. (2017). Combining H/D Exchange Mass Spectrometry and Computational Docking to Derive the Structure of Protein–Protein Complexes. Biochemistry.

[136] Kim S.S., Seffernick J.T., Lindert S. (2018). Accurately Predicting Disordered Regions of Proteins Using Rosetta ResidueDisorder Application. J. Phys. Chem. B.

[137] Seffernick J.T., Ren H., Kim S.S., Lindert S. (2019). Measuring Intrinsic Disorder and Tracking Conformational Transitions Using Rosetta ResidueDisorder. J. Phys. Chem. B.

[138] Hass M.A., Ubbink M. (2014). Structure Determination of Protein–Protein Complexes with Long-Range Anisotropic Paramagnetic NMR Restraints. Curr. Opin. Struct. Biol..

[139] Nitsche C., Otting G. (2017). Pseudocontact Shifts in Biomolecular NMR Using Paramagnetic Metal Tags. Prog. Nucl. Magn. Reson. Spectrosc..

[140] Softley C.A., Bostock M.J., Popowicz G.M., Sattler M. (2020). Paramagnetic NMR in Drug Discovery. J. Biomol. NMR.

[141] Ravera E., Gigli L., Fiorucci L., Luchinat C., Parigi G. (2022). The Evolution of Paramagnetic NMR as a Tool in Structural Biology. Phys. Chem. Chem. Phys..

[142] Müntener T., Joss D., Häussinger D., Hiller S. (2022). Pseudocontact Shifts in Biomolecular NMR Spectroscopy. Chem. Rev..

[143] Allegrozzi M., Bertini I., Janik M.B.L., Lee Y.-M., Liu G., Luchinat C. (2000). Lanthanide-Induced Pseudocontact Shifts for Solution Structure Refinements of Macromolecules in Shells up to 40 Å from the Metal Ion. J. Am. Chem. Soc..

[144] Pilla K.B., Leman J.K., Otting G., Huber T. (2015). Capturing Conformational States in Proteins Using Sparse Paramagnetic NMR Data. PLoS ONE.

[145] Hartlmüller C., Günther J.C., Wolter A.C., Wöhnert J., Sattler M., Madl T. (2017). RNA Structure Refinement Using NMR Solvent Accessibility Data. Sci. Rep..

[146] Loquet A., Sgourakis N.G., Gupta R., Giller K., Riedel D., Goosmann C., Griesinger C., Kolbe M., Baker D., Becker S. (2012). Atomic Model of the Type III Secretion System Needle. Nature.

[147] Das R., Andre I., Shen Y., Wu Y., Lemak A., Bansal S., Arrowsmith C.H., Szyperski T., Baker D. (2009). Simultaneous Prediction of Protein Folding and Docking at High Resolution. Proc. Natl. Acad. Sci. USA.

[148] Demers J.P., Habenstein B., Loquet A., Kumar Vasa S., Giller K., Becker S., Baker D., Lange A., Sgourakis N.G. (2014). High-Resolution Structure of the Shigella Type-III Secretion Needle by Solid-State NMR and Cryo-Electron Microscopy. Nat. Commun..

[149] Morag O., Sgourakis N.G., Baker D., Goldbourt A. (2015). The NMR–Rosetta Capsid Model of M13 Bacteriophage Reveals a Quadrupled Hydrophobic Packing Epitope. Proc. Natl. Acad. Sci. USA.

[150] Chou H.-T., Apelt L., Farrell D.P., White S.R., Woodsmith J., Svetlov V., Goldstein J.S., Nager A.R., Li Z., Muller J. (2019). The Molecular Architecture of Native BBSome Obtained by an Integrated Structural Approach. Structure.

[151] Mashtalir N., Suzuki H., Farrell D.P., Sankar A., Luo J., Filipovski M., D’Avino A.R., St. Pierre R., Valencia A.M., Onikubo T. (2020). A Structural Model of the Endogenous Human BAF Complex Informs Disease Mechanisms. Cell.

[152] Rives A., Meier J., Sercu T., Goyal S., Lin Z., Liu J., Guo D., Ott M., Zitnick C.L., Ma J. (2021). Biological Structure and Function Emerge from Scaling Unsupervised Learning to 250 Million Protein Sequences. Proc. Natl. Acad. Sci. USA.

[153] Wu R., Ding F., Wang R., Shen R., Zhang X., Luo S., Su C., Wu Z., Xie Q., Berger B. (2022). High-Resolution de Novo Structure Prediction from Primary Sequence. bioRxiv.

[154] Zweckstetter M. (2021). NMR Hawk-Eyed View of AlphaFold2 Structures. Protein Sci..

[155] Tejero R., Huang Y.J., Ramelot T.A., Montelione G.T. (2022). AlphaFold Models of Small Proteins Rival the Accuracy of Solution NMR Structures. Front. Mol. Biosci..

[156] Varadi M., Anyango S., Deshpande M., Nair S., Natassia C., Yordanova G., Yuan D., Stroe O., Wood G., Laydon A. (2022). AlphaFold Protein Structure Database: Massively Expanding the Structural Coverage of Protein-Sequence Space with High-Accuracy Models. Nucleic Acids Res..

[157] Stahl K., Graziadei A., Dau T., Brock O., Rappsilber J. (2023). Protein Structure Prediction with in-Cell Photo-Crosslinking Mass Spectrometry and Deep Learning. Nat. Biotechnol..

[158] Watson J.L., Juergens D., Bennett N.R., Trippe B.L., Yim J., Eisenach H.E., Ahern W., Borst A.J., Ragotte R.J., Milles L.F. (2022). Broadly Applicable and Accurate Protein Design by Integrating Structure Prediction Networks and Diffusion Generative Models. bioRxiv.

[159] del Alamo D., Sala D., Mchaourab H.S., Meiler J. (2022). Sampling Alternative Conformational States of Transporters and Receptors with AlphaFold2. eLife.

[160] Stein R.A., Mchaourab H.S. (2022). SPEACH_AF: Sampling Protein Ensembles and Conformational Heterogeneity with Alphafold2. PLoS Comput. Biol..

[161] Heo L., Feig M. (2022). Multi-State Modeling of G-Protein Coupled Receptors at Experimental Accuracy. Proteins Struct. Funct. Bioinform..

[162] Laurents D.V. (2022). AlphaFold 2 and NMR Spectroscopy: Partners to Understand Protein Structure, Dynamics and Function. Front. Mol. Biosci..

[163] Sauerwein A.C., Hansen D.F., Berliner L. (2015). Relaxation Dispersion NMR Spectroscopy. Protein NMR: Modern Techniques and Biomedical Applications.

[164] Fenwick R.B., Oyen D., van den Bedem H., Dyson H.J., Wright P.E. (2021). Modeling of Hidden Structures Using Sparse Chemical Shift Data from NMR Relaxation Dispersion. Biophys. J..

[165] Hass M.A.S., Liu W.-M., Agafonov R.V., Otten R., Phung L.A., Schilder J.T., Kern D., Ubbink M. (2015). A Minor Conformation of a Lanthanide Tag on Adenylate Kinase Characterized by Paramagnetic Relaxation Dispersion NMR Spectroscopy. J. Biomol. NMR.

[166] Ma R.S., Li Q.F., Wang A.D., Zhang J.H., Liu Z.J., Wu J.H., Su X.C., Ruan K. (2016). Determination of Pseudocontact Shifts of Low-Populated Excited States by NMR Chemical Exchange Saturation Transfer. Phys. Chem. Chem. Phys..

[167] Vallurupalli P., Kay L.E. (2006). Complementarity of Ensemble and Single-Molecule Measures of Protein Motion: A Relaxation Dispersion NMR Study of an Enzyme Complex. Proc. Natl. Acad. Sci. USA.

[168] Zintsmaster J.S., Wilson B.D., Peng J.W. (2008). Dynamics of Ligand Binding from 13C NMR Relaxation Dispersion at Natural Abundance. J. Am. Chem. Soc..

[169] Ward J.J., Sodhi J.S., McGuffin L.J., Buxton B.F., Jones D.T. (2004). Prediction and Functional Analysis of Native Disorder in Proteins from the Three Kingdoms of Life. J. Mol. Biol..

[170] Pentony M.M., Jones D.T. (2010). Modularity of Intrinsic Disorder in the Human Proteome. Proteins Struct. Funct. Bioinform..

[171] Wilson C.J., Choy W.-Y., Karttunen M. (2022). AlphaFold2: A Role for Disordered Protein/Region Prediction?. IJMS.

[172] Martin E.W., Hopkins J.B., Mittag T., Keating C.D. (2021). Chapter Seven—Small-Angle X-Ray Scattering Experiments of Monodisperse Intrinsically Disordered Protein Samples Close to the Solubility Limit. Methods in Enzymology.

[173] Murthy A.C., Fawzi N.L. (2020). The (Un)Structural Biology of Biomolecular Liquid-Liquid Phase Separation Using NMR Spectroscopy. J. Biol. Chem..

[174] Wang R.Y.-R., Han Y., Krassovsky K., Sheffler W., Tyka M., Baker D. (2011). Modeling Disordered Regions in Proteins Using Rosetta. PLoS ONE.

[175] Kleiger G., Saha A., Lewis S., Kuhlman B., Deshaies R.J. (2009). Rapid E2–E3 Assembly and Disassembly Enable Processive Ubiquitylation of Cullin-RING Ubiquitin Ligase Substrates. Cell.

[176] Ferrie J.J., Petersson E.J. (2020). A Unified De Novo Approach for Predicting the Structures of Ordered and Disordered Proteins. J. Phys. Chem. B.

[177] Christoffer C., Kihara D., Kihara D. (2020). IDP-LZerD: Software for Modeling Disordered Protein Interactions. Protein Structure Prediction.

[178] Scheres S.H., Zhang W., Falcon B., Goedert M. (2020). Cryo-EM Structures of Tau Filaments. Curr. Opin. Struct. Biol..

[179] Shi Y., Zhang W., Yang Y., Murzin A.G., Falcon B., Kotecha A., van Beers M., Tarutani A., Kametani F., Garringer H.J. (2021). Structure-Based Classification of Tauopathies. Nature.

[180] Paravastu A.K., Leapman R.D., Yau W.-M., Tycko R. (2008). Molecular Structural Basis for Polymorphism in Alzheimer’s β-Amyloid Fibrils. Proc. Natl. Acad. Sci. USA.

[181] Lu J.-X., Qiang W., Yau W.-M., Schwieters C.D., Meredith S.C., Tycko R. (2013). Molecular Structure of β-Amyloid Fibrils in Alzheimer’s Disease Brain Tissue. Cell.

[182] Sgourakis N.G., Yau W.-M., Qiang W. (2015). Modeling an In-Register, Parallel “Iowa” Aβ Fibril Structure Using Solid-State NMR Data from Labeled Samples with Rosetta. Structure.

[183] Tuttle M.D., Comellas G., Nieuwkoop A.J., Covell D.J., Berthold D.A., Kloepper K.D., Courtney J.M., Kim J.K., Barclay A.M., Kendall A. (2016). Solid-State NMR Structure of a Pathogenic Fibril of Full-Length Human α-Synuclein. Nat. Struct. Mol. Biol..

